# Supplementary material for: A Cell-Autonomous Oncosuppressive Role of Human RNASET2 Affecting ECM-Mediated Oncogenic Signaling
Source: Cancers (Basel). 2019 Feb 22;11(2):255. doi: 10.3390/cancers11020255 (PMC6406318; doi:10.3390/cancers11020255)
Supplement: Supplementary file 1 [file cancers-11-00255-s001.pdf]

cancers

Supplementary Materials: A Cell-Autonomous Oncosuppressive Role of Human RNASET2 Affecting ECM-Mediated Oncogenic Signaling

Francesca Roggiani, Cristina Riva, Francesco Raspagliesi, Giovanni Porta, Roberto Valli, Roberto Taramelli, Francesco Acquati, Delia Mezzanzanica and Antonella Tomassetti

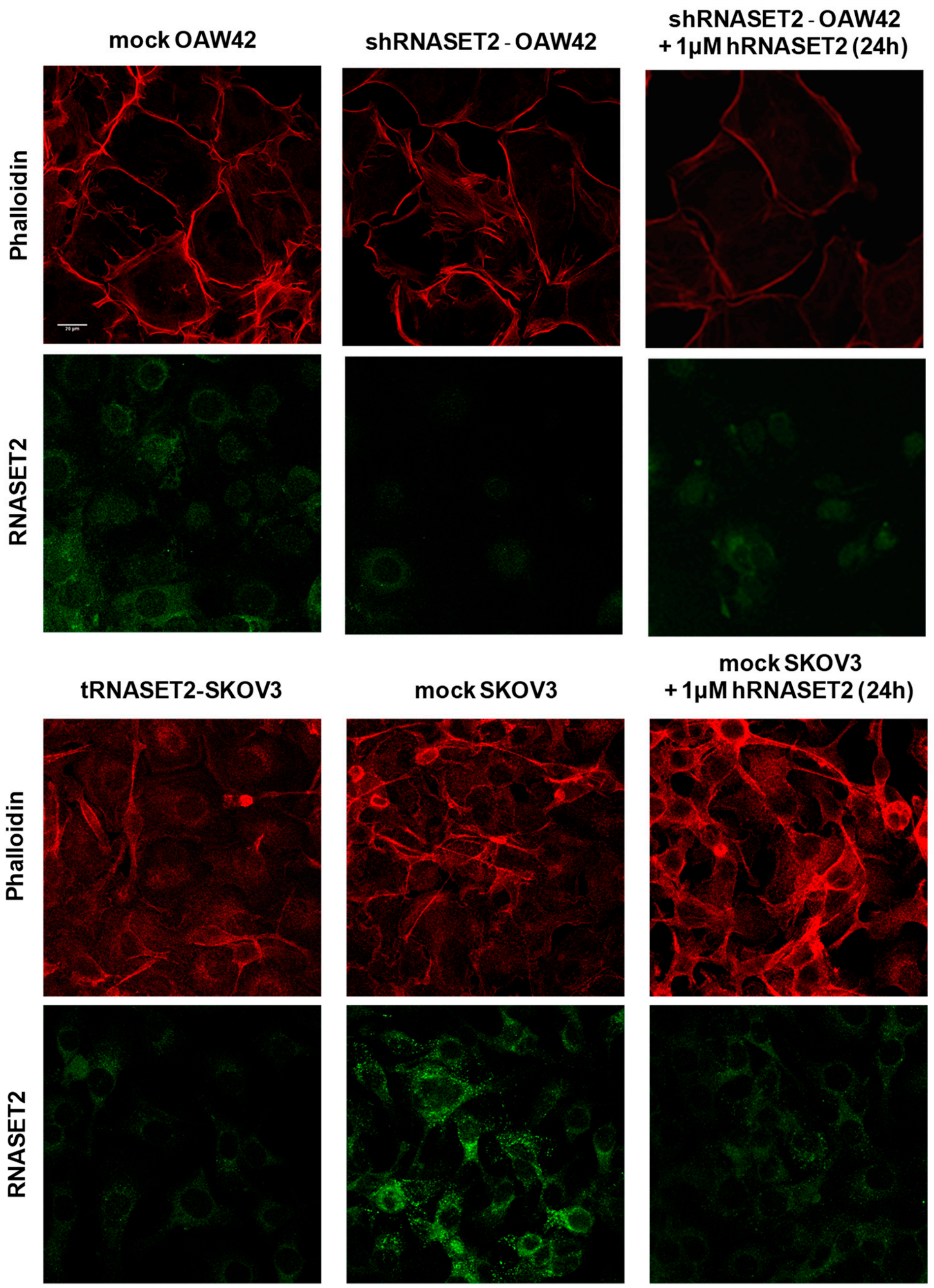

(a)

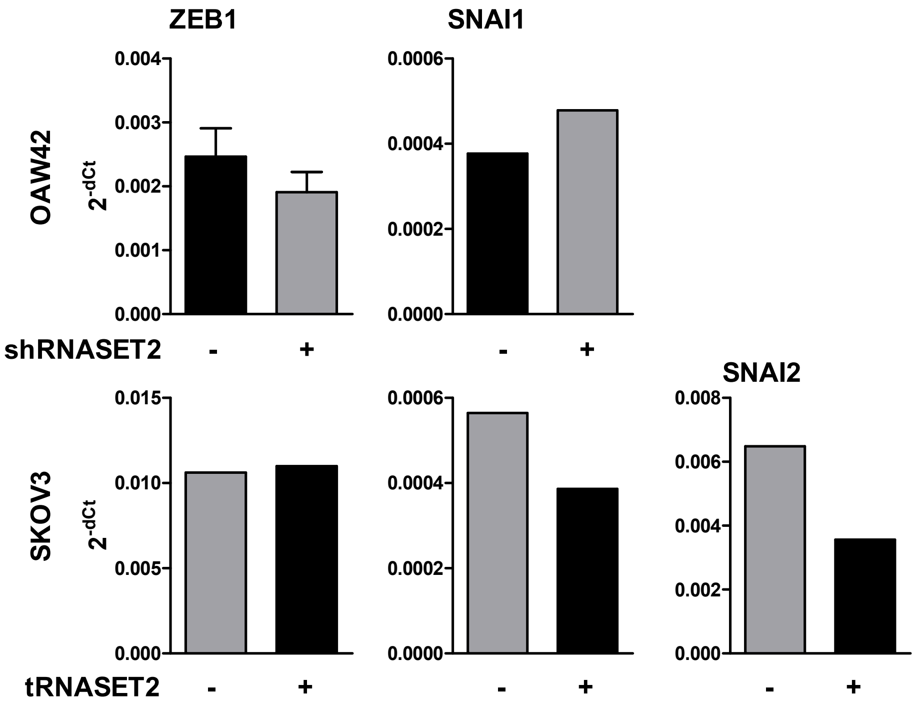

(**b**)

Figure S1. (**a**) Confocal IF analysis on fixed mock- or shRNASET2-OAW42 and mock- or Table 2. cells treated or not with hRNASET2 (1 μM) for 24 h. Staining was performed with Abs reported on the left. (**b**) Real-time RT-PCR showing the transcript levels of ZEB1, SNAI1 and SNAI2 in mock/shRNASET2-OAW42 and mock/tRNASET2-SKOV3 cells. Results are presented as relative expression normalized to GAPDH mRNA levels from three experiments. Error bars, SD.

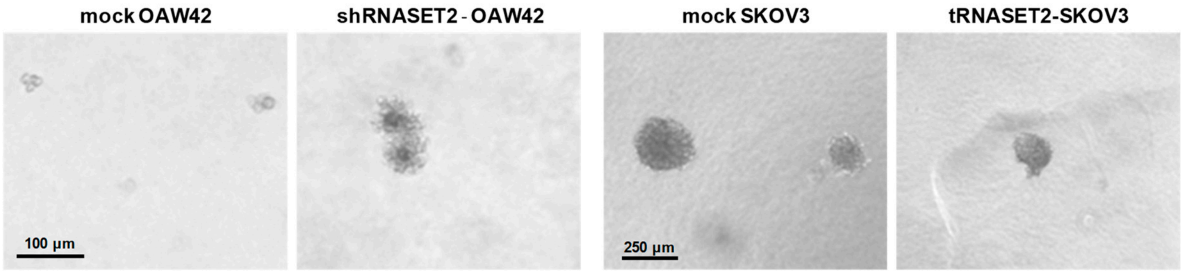

(**a**)

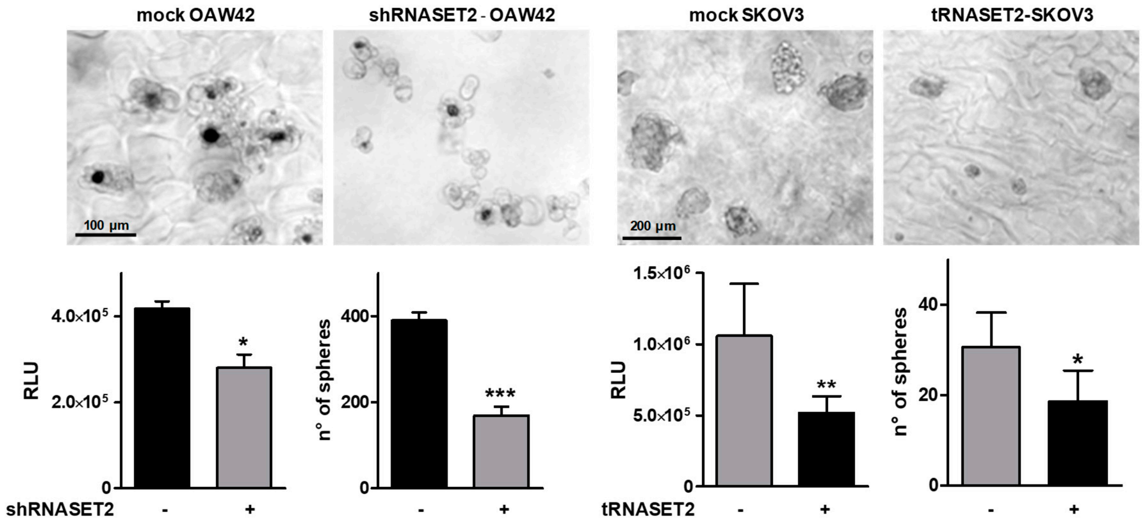

(**b**)

Figure S2. (**a**) Representative phase contrast images of the clonogenic assay described in Figure 3b. (**b**) Three-dimensional Algimatrix™ cultures of mock- or shRNASET2-OAW42 (left panel) and mock- or tRNASET2-SKOV3 cells (right panel) grown for 10 days. Upper panel: representative phase contrast images of cell spheres. Lower panel: graphs reporting the relative luminescence units (RLU) measured by CellTiter-Glo® Luminescent Cell viability assay (Promega) or as the number of spheres/well. Asterisks indicate significant values (* $p < 0.05$, ** $p < 0.01$ and *** $p < 0.001$,). Error bars, SD.

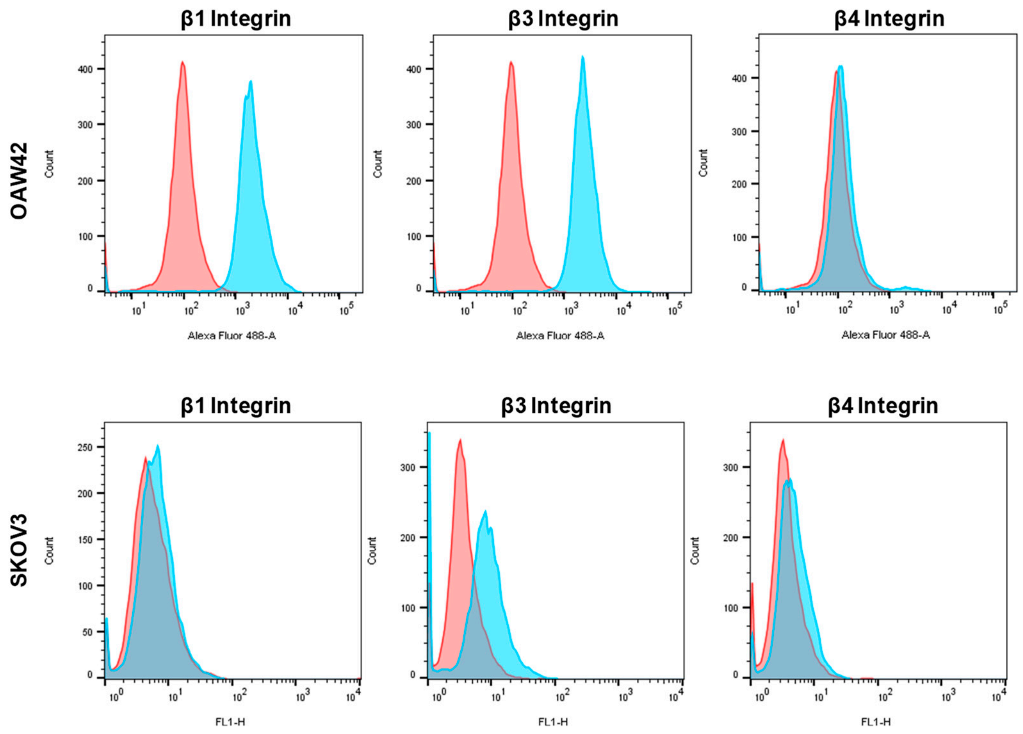

(**a**)

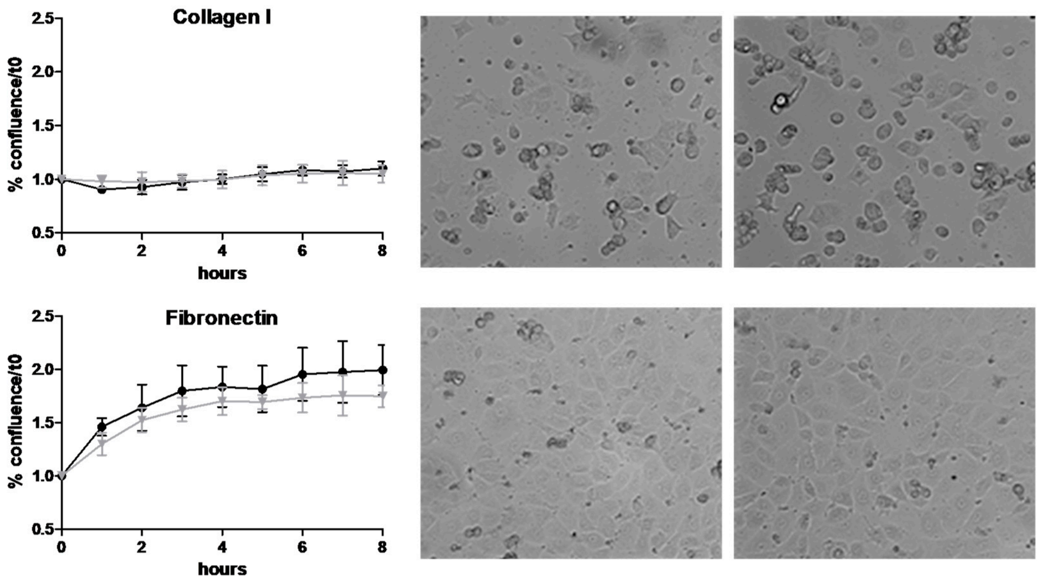

(**b**)

Figure S3. (**a**) FACS analysis performed with anti-β1, β3 and β4 integrin Abs on OAW42 (upper panel) and SKOV3 (lower panel) cells. Secondary Abs were used as negative control. (**b**) Adhesion on ECM proteins of mock- (black line) and shRNASET2 (grey line) OAW42 cells. Left panels: cells were monitored for up to 8 h, measuring cell confluence with JuLI™ Stage microscope and software (NanoEntek). Right panel: representative phase contrast imaged captured by the JuLI™ Stage microscope after 8 h.

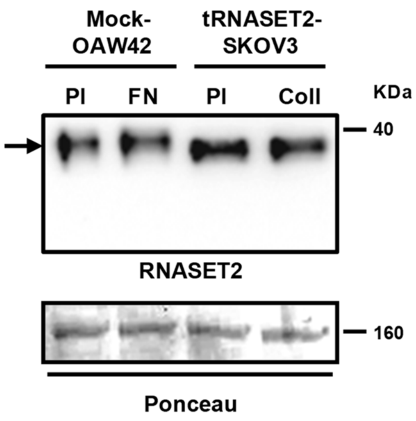

Figure S4. Upper panel: immunoblotting with anti-RNASET2 performed on three days conditioned media of RNASET2-expressing mock-OAW42 and tRNASET2-SKOV3 cells. The arrow highlights the 36 KDa band of secreted RNASET2. Lower panel: gel loading visualized as ponceau staining of the membrane.

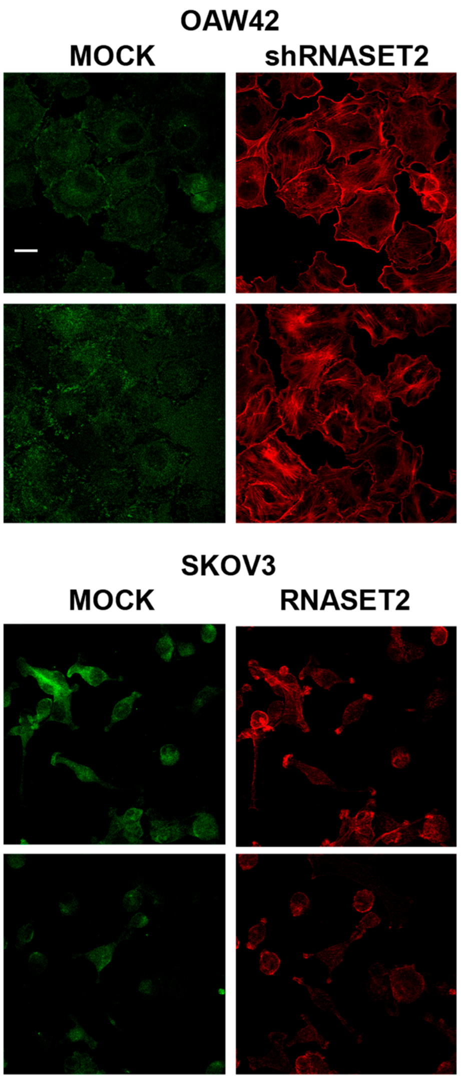

Figure S5. Images with single Ab of the confocal IF showed in Figure 4c. The white empty boxes highlight the images reported in Figure 4c. Green, anti-phosphorylated paxillin; red, phalloidin. Bar 20 µm.

Table S1. List of used antibodies.

Primary antibodies	Source	Catalog #	Dilution for IHC	Dilution for WB	Dilution for IF	Dilution for FACS
RNASET2	[1]		5 μg/mL	1:500	1:200	
E-cadherin	ThermoFisher Scientific	131700		1:400	1:500	
N-cadherin	ThermoFisher Scientific	180224		1:200	1:100	
Vimentin	Novocastra	Ncl-l-vim-v9		1:200		
Cytokeratin 8/18	Novocastra	Ncl-l-5d3		1:200		
β-actin	SIGMA-ALDRICH	A2066		1:400		
Phalloidin	ThermoFisher Scientific	A22283			1:1000	
β1 Integrin	Santa Cruz Biotechnology	Sc-6622				10 μg/mL
β3 Integrin	Origene	TA320397				10 μg/mL
B4 Integrin	Santa Cruz Biotechnology	Sc-13127				10 μg/mL
P-Paxillin (Tyr118)	ThermoFisher Scientific	44722G			1:50	
P-SRC (Tyr416)	Cell Signaling	2101s		1:500		
SRC	Santa Cruz Biotechnology	Sc-19		1:500		
P-AKT (Ser473)	Cell Signaling	9271s		1:1000		
AKT	Cell Signaling	9272s		1:500		
P-MAPK (Thr202/Tyr204)	Cell Signaling	9101s		1:1000		
ERK 1/2	Santa Cruz Biotechnology	Sc-154/Sc-93		1:200		

References

1 Campomenosi, P.; Cinquetti, R.; Tallarita, E.; Lindqvist, C.; Raimondi, I.; Grassi, P.; Näsman, J.; Dell, A.; Haslam, S.M.; Taramelli, R.; et al. Comparison of the baculovirus-insect cell and Pichia pastoris heterologous systems for the expression of the human tumor suppressor protein RNASET2. *Biotechnol. Appl. Biochem.* **2011**, *58*, 39–49.

© 2019 by the authors. Licensee MDPI, Basel, Switzerland. This article is an open access article distributed under the terms and conditions of the Creative Commons Attribution (CC BY) license (http://creativecommons.org/licenses/by/4.0/).
